# The Interaction of Artificial Intelligence and Social Media in Dermatology

**DOI:** 10.1111/jocd.71035

**Published:** 2026-07-01

**Authors:** Mohamad Goldust

**Affiliations:** ^1^ Department of Dermatology University of Illinois at Chicago Chicago Illinois USA

**Keywords:** artificial intelligence, dermatology, digital health, social media, teledermatology

Dear Editor,

The integration of artificial intelligence (AI) with social media is reshaping dermatology by creating new opportunities for education, outreach, and early disease detection. As a visually driven specialty, dermatology is particularly well‐suited for image‐based AI applications and digital dissemination. However, alongside these advantages, there are significant concerns related to diagnostic accuracy, misinformation, bias, and regulatory oversight that require urgent attention [1].

AI‐enabled tools are increasingly integrated into platforms such as Instagram, TikTok, and Snapchat, where they facilitate automated skin analysis, lesion triage, and cosmetic simulations. Advances in deep learning, including convolutional neural networks and vision transformers, have shown dermatologist‐level performance in controlled settings for tasks such as classifying pigmented lesions and grading acne. However, real‐world performance remains inconsistent. Many commercially available tools lack external validation, standardized datasets, and regulatory clearance as Software as a Medical Device (SaMD). As a result, users may misinterpret outputs from non‐medical algorithms as clinical advice, increasing the risk of delayed diagnoses or inappropriate self‐treatment [2]. Importantly, a distinction should be made between consumer‐facing AI applications designed primarily for wellness, cosmetic visualization, or general educational purposes and clinically validated AI tools that undergo regulatory review and are intended to support medical decision‐making. Failure to differentiate these categories may contribute to public misunderstanding and inappropriate reliance on non‐medical platforms for diagnostic guidance.

A major limitation of these tools is the quality and context of user‐generated images. Unlike curated clinical datasets, social media images are often affected by poor lighting, filters, occlusion, and a lack of clinical metadata, such as lesion history or symptoms. Algorithm performance may decline significantly under these conditions. Furthermore, the “black box” nature of many AI systems limits interpretability, which reduces clinician trust and complicates medico‐legal accountability.

Ethical and equity concerns further complicate this landscape. Algorithmic bias is a critical issue, with reduced diagnostic accuracy reported for individuals with darker skin types due to underrepresentation in training datasets. This risks exacerbating disparities in skin cancer detection and inflammatory conditions. Additionally, AI tools often require users to upload sensitive images, raising concerns about informed consent, data ownership, and compliance with privacy regulations like HIPAA and GDPR [3].

Beyond diagnostics, AI is transforming how dermatological information is created and disseminated. Generative AI and large language models can rapidly produce educational content, increasing efficiency for dermatologists. However, they also allow non‐experts and influencers to generate authoritative‐sounding but potentially inaccurate information. Studies suggest that over 30%–40% of dermatology‐related social media content contains misleading or non‐evidence‐based claims, particularly regarding acne treatments and cosmetic procedures. The algorithm‐driven amplification of this content further accelerates the spread of misinformation [4].

Conversely, AI offers powerful tools for public health surveillance. Machine learning and natural language processing can analyze large‐scale social media data to identify emerging dermatological trends, monitor adverse reactions, and detect misinformation in real time. These insights can support targeted, evidence‐based interventions led by dermatologists and professional organizations.

To maximize benefits while minimizing risks, robust multidisciplinary control is essential. Regulatory agencies should establish clearer distinctions between medical‐grade AI and consumer applications, requiring clinical validation, transparency in training datasets, and post‐market surveillance. Dermatology societies should develop guidelines for the ethical use of AI on social media, including standards for disclosure, patient consent, and conflict of interest. Improving the representation of diverse skin types in training datasets is also critical for ensuring equitable performance [5].

Educational initiatives are equally important. Dermatologists must develop digital literacy and AI competency, whereas patients should be empowered to critically evaluate online content. Strengthening the presence of board‐certified dermatologists on social media may help counter misinformation and enhance public trust. In addition, strategies to improve AI literacy among both clinicians and patients should include educational workshops, integration of AI‐related topics into medical training curricula, and public awareness campaigns focused on the strengths and limitations of AI‐generated dermatologic information. Such measures may help users better distinguish evidence‐based recommendations from misleading or non‐validated online content.

In conclusion, the connection between AI and social media presents both a transformative opportunity and a significant challenge in dermatology. Although these technologies can expand access and improve education, their unregulated use risks compromising diagnostic accuracy, patient safety, and equity. A balanced, evidence‐based approach grounded in ethical principles and active dermatologic leadership is essential to ensure that digital innovation ultimately enhances patient care.

## Funding

The author has nothing to report.

## Ethics Statement

The author has nothing to report.

## Conflicts of Interest

The author declares no conflicts of interest.

## Data Availability

The author has nothing to report.

## References

[1] S. P. Kololgi and C. S. Lahari , “Harnessing the Power of Artificial Intelligence in Dermatology: A Comprehensive Commentary,” Indian Journal of Dermatology 68, no. 6 (2023): 678–681. 38371574 10.4103/ijd.ijd_581_23 PMC10868991

[2] O. Uwishema , M. Ghezzawi , N. Charbel , et al., “Diagnostic Performance of Artificial Intelligence for Dermatological Conditions: A Systematic Review Focused on Low‐ and Middle‐Income Countries to Address Resource Constraints and Improve Access to Specialist Care,” International Journal of Emergency Medicine 18, no. 1 (2025): 172. 41023774 10.1186/s12245-025-00975-4 PMC12481837

[3] J. W. Tjiu and C. F. Lu , “Equity and Generalizability of Artificial Intelligence for Skin‐Lesion Diagnosis Using Clinical, Dermoscopic, and Smartphone Images: A Systematic Review and Meta‐Analysis,” Medicina (Kaunas, Lithuania) 61, no. 12 (2025): 2186. 41470188 10.3390/medicina61122186 PMC12735087

[4] D. du Crest , M. Madhumita , W. Enbiale , et al., “AI and Digital Tools in Dermatology: Addressing Access and Misinformation,” JMIR Dermatology 9 (2026): e79044. 41719483 10.2196/79044 PMC12923100

[5] M. Goldust and J. M. Grant‐Kels , “Professional Standards and Regulations for the Use of Artificial Intelligence in Dermatology,” International Journal of Dermatology 63, no. 10 (2024): e274–e275. 38727097 10.1111/ijd.17235

